# Sex Hormone Candidate Gene Polymorphisms Are Associated with Endometriosis

**DOI:** 10.3390/ijms232213691

**Published:** 2022-11-08

**Authors:** Ilya Golovchenko, Boris Aizikovich, Oleg Golovchenko, Evgeny Reshetnikov, Maria Churnosova, Inna Aristova, Irina Ponomarenko, Mikhail Churnosov

**Affiliations:** 1 Department of Medical Biological Disciplines, Belgorod State University, 308015 Belgorod, Russia; 2 Department of Fundamental Medicine, Novosibirsk State University, 630090 Novosibirsk, Russia; 3 Department of Obstetrics and Gynecology, Belgorod State University, 308015 Belgorod, Russia

**Keywords:** sex hormones, endometriosis, SNP, association

## Abstract

The present study was designed to examine whether sex hormone polymorphisms proven by GWAS are associated with endometriosis risk. Unrelated female participants totaling 1376 in number (395 endometriosis patients and 981 controls) were recruited into the study. Nine single-nucleotide polymorphisms (SNPs) which GWAS correlated with circulating levels of sex hormones were genotyped using a TaqMan allelic discrimination assay. FSH-lowering, and LH- and testosterone-heightening polymorphisms of the *FSHB* promoter (allelic variants A rs11031002 and C rs11031005) exhibit a protective effect for endometriosis (OR = 0.60–0.68). By contrast, the TT haplotype loci that were GWAS correlated with higher FSH levels and lower LH and testosterone concentrations determined an increased risk for endometriosis (OR = 2.03). Endometriosis-involved epistatic interactions were found between eight loci of sex hormone genes (without rs148982377 *ZNF789*) within twelve genetic simulation models. In silico examination established that 8 disorder-related loci and 80 proxy SNPs are genome variants affecting the expression, splicing, epigenetic and amino acid conformation of the 34 genes which enrich the organic anion transport and secondary carrier transporter pathways. In conclusion, the present study showed that sex hormone polymorphisms proven by GWAS are associated with endometriosis risk and involved in the molecular pathophysiology of the disease due to their functionality.

## 1. Introduction

Endometriosis is one of the most common infertility-associated disorders, affecting 5–10% women of reproductive age and 35–50% of infertile women overall [1,2]. The disease affects approximately 176–179 million women worldwide [1,3]. Endometriosis is defined by the presence of endometrial tissue (glands/stromal) outside the uterus (organs affected include the peritoneum, ovary, fallopian tubes, etc.) [2,3,4]. The ectopic endometrial tissue goes through menstrual cycle changes like normally located endometrium, but with no means of exiting the body [4]. Several clinical manifestations including chronic pelvic pain, dysmenorrhea (heavy menstrual bleeding, etc.), infertility, and dyspareunia characterize the disorder [1,2]. There are wide-ranging socio-economic burdens associated with endometriosis—for the sufferers, their families, and for society at large [2,3,5].

The molecular mechanisms of endometriosis remain highly enigmatic and largely unknown [6,7]. The disorder biology is complex (multifactorial), involving multiple hormonal, inflammatory, genetic, immunological and environmental factors as causes [2]. The genetic factors play an important role in endometriosis etiology [6,8,9,10,11]. A twin-based study showed that genetic influences on the development of endometriosis are quite considerable and amount to 47% [8]. Lee et al. evaluated the SNP-heritability of endometriosis as 26% [9]. In a genome-wide association study (GWAS), Sapkota et al. revealed that 5.19% of the endometriosis variability is determined by 19 GWAS-significant loci [6]. Thus, the indicator of the contribution of hereditary factors to the development of endometriosis, obtained on the basis of known GWAS-significant polymorphisms (≈5%), is almost 10 times less than the similar indicator obtained from the twin-based data (47%). These data demonstrate that at this moment, it is clear that genetic factors have a great impact on this trait, and also clear that little is known about specific genetic variants that contribute to the endometriosis variation [11]. Hence, many genetic variants contributing to endometriosis variation have yet to be elicited and further research is needed to understand the genetic complexity underlying endometriosis [11].

Endometriosis is a hormone-dependent gynecological disease, associated with sex hormones (oestradiol, progesterone, follicle-stimulating hormone (FSH), etc.) [3,4,7]. Numerous associative studies, including GWAS, have shown the relationship between endometriosis and polymorphism of sex hormone genes (*FN1*, *FSHB*, *ESR1*, *CCDC170*, *LHCGR*, *SYNE1*, etc.) [5,6,10,12,13]. At the same time, there are GWAS data on the correlation of a number of SNPs with the level of several sex hormones (estradiol, SHBG, FAI, testosterone, DHEAS, LH, FSH, progesterone) [14,15,16,17]. It can be assumed that GWAS-significant polymorphisms at the level of sex hormones may be involved in the biology of endometriosis. It should be noted that in previous studies only some of these polymorphic loci of the *FSHB* promoter (rs11031002 and rs11031005) and strongly linked SNPs (rs74485684, rs11031010r, s10835638, rs1782507, rs555621) have had demonstrated associations with endometriosis [5,6,10,12,18]. Conversely, mendelian randomization analysis of the endometriosis and sex hormones GWAS data [6 and 16 respectively] performed by Garitazelaia et al. did not show reliable causal relationships between endometriosis and reproductive hormone levels [18]. The existing ambiguity of this issue dictates the need to continue research on this problem.

The present study was undertaken to investigate whether sex hormone gene polymorphisms proved by GWAS are associated with endometriosis risk.

## 2. Results

The frequency genotypes and alleles of the considered SNPs in endometriosis/control groups were associated with HWE (P_bonferroni_ > 0.05/9 = 0.0055) (Supplementary Table S1).

Among the nine SNPs identified, only the two located in the *FSHB* chain promoter (37kb 5′ of *FSHB* rs11031002 T>A and 26kb 5′ of *FSHB* rs11031005 T>C) seem to have a significant impact on endometriosis risk (Table 1). We discovered lower risk of endometriosis among A rs11031002 and C rs11031005 carriers (Table 1). We observed this pattern in almost all the studied genetic models: allelic (OR = 0.68; 95% CI = 0.52–0.90; P = 0.006; P_permutation_ = 0.010 and OR = 0.66; 95% CI = 0.50–0.87; P = 0.003; P_permutation_ = 0.004 respectively), additive (OR = 0.64; 95% CI = 0.47–0.85; *Power* = 89.34%; P = 0.003; P_permutation_ = 0.003 and OR = 0.65; 95% CI = 0.49–0.87; *Power* = 87.26%; P = 0.004; P_permutation_ = 0.004 respectively) and dominant (OR = 0.60; 95% CI = 0.43–0.82; *Power* = 92.03%; P = 0.002; P_permutation_ = 0.002 and OR = 0.65; 95% CI = 0.49–0.92; *Power* = 81.92%; P = 0.009; P_permutation_ = 0.010 respectively).

Additionally, we also found evidence which suggests that haplotype variants of the *FSHB* subunit might be implicated in the development of endometriosis (Table 2). Higher prevalence of TT haplotype carrier [rs11031002-rs11031005] was observed in endometriosis than in the control group (0.898 vs. 0.842; OR = 2.03; P = 2 × 10^−6^; P_permutation_ = 0.001), and conversely, AT and TC haplotypes were considerably less frequent in patients with regard to controls (0.008 vs. 0.022; OR = 0.09; P = 1 × 10^−6^; P_permutation_ = 0.001 and 0.007 vs. 0.023; OR = 0.16; P = 2 × 10^−4^; P_permutation_ = 0.001) (Table 2).

In the next step of our work, we performed a simulation of interaction of non-allelic genes (epistasis interaction) where the effect of the studied SNPs/genes combine to produce endometriosis. The permissible MB-MDR analysis identified twelve epistasis interaction models causal for the disease (Table 3). Among eight loci included in these simulation genetic models (only rs148982377 *ZNF789* was excluded), two SNPs (*FSHB*-rs11031002 and rs11031005) were implicated in a significant number (12 (100%) and 8 (67%), respectively) of epistasis models.

Three-way and four-way simulation genetic models such as rs11031002 *FSHB* × rs117585797 *ANO2* × rs11031005 *FSHB* and rs11031002 *FSHB* × rs117585797 *ANO2* − rs112295236 *SLC22A10* × rs11031005 *FSHB* had the maximum effects for the disease (Wald parameters were equal to 36.04 and 35.70, respectively). The pair interaction between rs11031002 and rs11031005 *FSHB* subunit had predominant effects for the vast majority (2/3) of the simulation genetic models under consideration (Wald statistic for this pair interaction was equal to 32.68). Genotypes epistasis interaction such as rs11031002 *FSHB* TA × rs112295236 *SLC22A10* CC × rs11031005 *FSHB* TT and rs11031002 *FSHB* TA × rs117585797 *ANO2* CC × rs112295236 *SLC22A10* CC × rs11031005 *FSHB* TT showed the most substantial protective effects on endometriosis risk (for both *beta* coefficients equal to −3.35, *p* = 0.000006) (Supplementary Table S2). Conversely, a genotype combination such as rs11031002 *FSHB* TT × rs11031005 *FSHB* TT demonstrated the most noticeable disorder risk effect (*beta* = 0.74 and *p* = 0.000002) (Supplementary Table S2).

**Figure 1:**
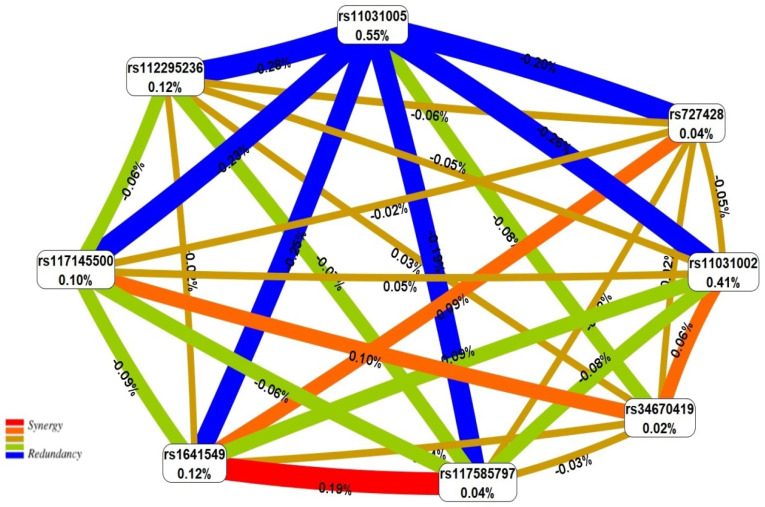
The entropy graph of the SNP×SNP interactions in endometriosis based on the MDR analysis. Positive values of entropy indicate synergistic interactions, while negative values indicate redundancy. The red and orange colors denote strong and moderate synergism, respectively; brown color denotes an independent effect; and green and blue colors denote moderate and strong antagonism, respectively.

The visual graphical presentation complex network of SNPs for endometriosis obtained by the entropy-based MDR method is presented in Figure 1. As seen in Figure 1, the “key” to the structure of the endometriosis complex SNP network are rs11031005 *FSHB* (causes −0.55% entropy), rs11031002 *FSHB* (−0.41%) and the paired antagonistic “dialogue” between rs11031005 *FSHB* and six other loci (rs11031002 *FSHB*, rs112295236 *SLC22A10*, rs117585797 *ANO2*, rs117145500 *CHD9*, rs727428 *SHBG*, rs1641549 *TP53*) (−0.19–−0.28%).

### In Silico SNP Analysis

Bioinformatics information implemented in HaploReg online resource (includes functionality annotations of the non-coding genome at variants on haplotype blocks) allowed us to identify the potential functions of SNPs of all eight loci associated with endometriosis and 80 strongly linked SNPs (Supplementary Table S3). Among these 88 genetic variants, the overwhelming majority of SNPs (82/88, 93.2%) have functionality (Supplementary Table S3): these are concentrated in important sequence regions such as “open” chromatin (hypersensitive sites to DNA nuclease) (11 SNPs); and gene transcription initiation and regulation regions (enhancers—16 SNPs, and promoters—8 SNPs) near *ZNF789*, *SLC22A24*, *SLC22A25*, *SLC22A10*, *TP53* and *SHBG* genes, potential gene expression regulatory regions (5′-UTR (1 SNP), 5′ (20 SNPs), 3′-UTR (1 SNP), 3′ (10 SNPs)) beside *FSHB*, *ZKSCAN5*, *SLC22A24*, *SLC22A25*, *CHD9*, *SHBG*, *RP11-467J12.4*; and *TP53* genes, specific transcriptional factors (79 SNPs) and regulatory proteins (4 SNPs) identification and binding sites near to *ANO2*, *FSHB*, *ZKSCAN5*, *SLC22A24*, *SLC22A25*, *CHD9*, *RP11-467J12.4*, *SHBG*, *SLC22A1*, *TP53* and *ZNF789* genes (Supplementary Table S3).

**Figure 2:**
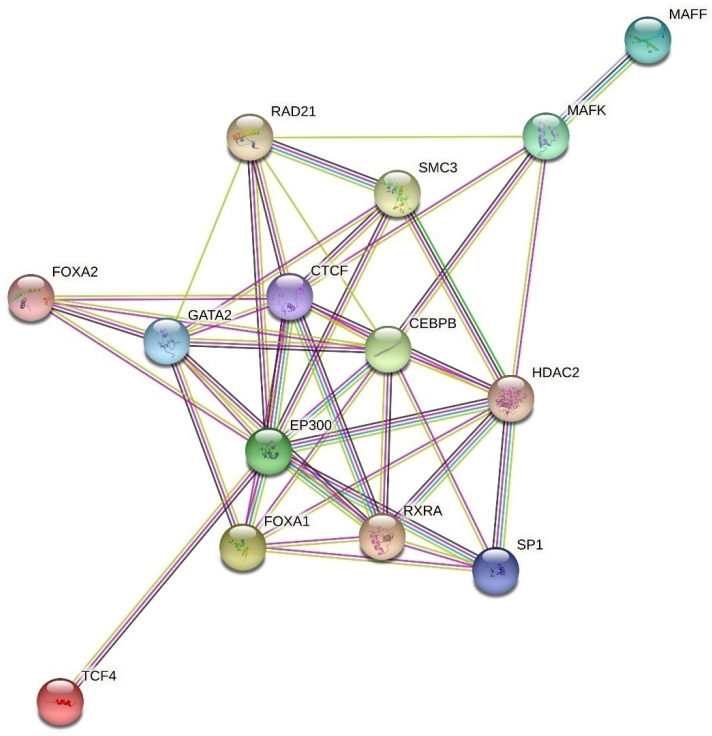
The interatomic network of the regulatory proteins involved in endometriosis inferred using STRING (https://string-db.org/ (accessed on 15 July 2022)).

Figure 2 shows an interatomic network involving fifteen regulatory proteins (SP1, FOXA1, FOXA2, P300, CFOS, GATA2, CTCF, RAD21, SMC3, CEBPB, HDAC2, MAFF, MAFK, RXRA, TCF4) whose potential binding sites were predicted in silico at the SNPs associated with endometriosis phenotypes and proxy SNPs (obtained using the STRING tool). Importantly, this set of endometriosis-related regulatory proteins enriched with the KEGG pathways (conducted by STRING) is also involved in the regulation of cell cycle (hsa04110, FDR = 0.0005), transcriptional misregulation in cancer (hsa05202, FDR = 0.001), thyroid hormone signaling pathway (hsa049193, FDR = 0.009), pathways in cancer (hsa05200, FDR = 0.03) and Notch signaling pathway (hsa04330, FDR = 0.04).

Based on the PolyPhen-2 (v2.2.2r406) data, among the examined polymorphisms, we found one non-synonymous locus—rs1042522 in *TP53* gene (this SNP is linked (r^2^ = 0.88) with a disease-involved locus rs1641549 *TP53*), which resulted in the same protein, Pro72Arg. The predicted category of this SNP was “BENIGN” (value of specific score parameter was equal to 0.083 with meaning sensitivity and specificity parameters equal to 0.93 and 0.85, respectively).

In our work, we accomplished comprehensive bioinformatics analysis tissue-specific expression (eQTL) and splicing (sQTL) quantitative traits using GTEx Consortium database (GTEx portal public resource) which made it possible to identify the linkage between variation in gene expression/splicing and endometriosis causal genetic polymorphisms and proxy loci (data are summarized in Supplementary Tables S4–S7).

We identified 25 genes whose expression level was associated with six endometriosis-related loci and 66 proxy SNPs with a significant tissue-specific component of gene expression signatures (Supplementary Tables S4 and S5). For instance, several SNPs such as rs34670419 *ZKSCAN5*, rs11031002 and rs11031005 *FSHB*, rs112295236 *SLC22A10*, rs727428 *SHBG*, and rs1641549 *TP53* are associated with gene expression changes in whole blood (*ZKSCAN5*, *TNFSF12*, *FXR2*, *TNFSF13*), thyroid (*GS1-259H13.2*, *ARL14EP*, *ARL14EP*, *EFNB3*), pituitary (*SHBG*), adipose (*ARL14EP*, *CYP3A7*, *EFNB3*), adrenal gland (*CYP3A7*), etc., thereby demonstrating their putative pathobiology involvement in endometriosis.

Furthermore, two causal loci for endometriosis (rs34670419 *ZKSCAN5* and rs727428 *SHBG*) and four polymorphisms that are in close linkage disequilibrium with them may modulate seven gene splicing in a tissue-specific manner (Supplementary Tables S6 and S7). Importantly, these splicing-active SNPs had molecular trait effects in the brain (substantia nigra) (*GPC2*), skeletal muscle (*AC113189.5*, *SAT2*, *FXR2*), whole blood (*TNFSF13*, *FXR2*), thyroid (*SAT2*, *FXR2*), adipose (*SAT2*, *AC113189.5*) and other organs which may suggest they play a significant role in the mechanisms of endometriosis development.

**Figure 3:**
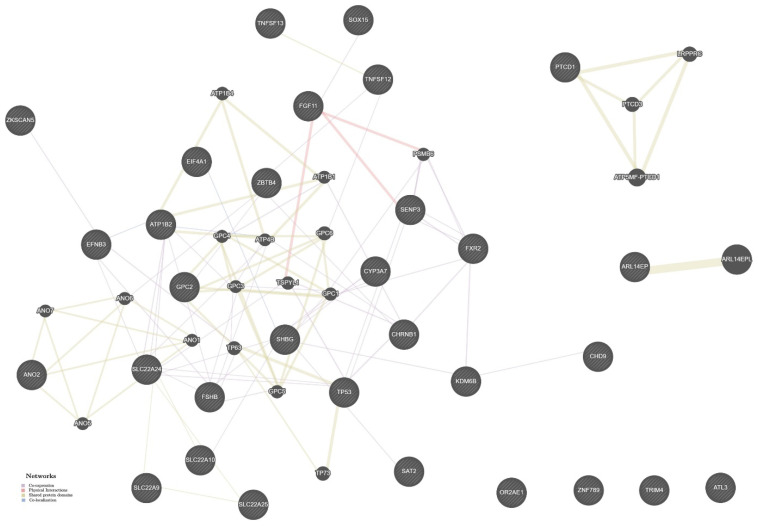
The interaction networks of the candidate genes for endometriosis in various tissues/organs inferred using GeneMANIA (http://genemania.org (accessed on 15 July 2022)). The candidate genes are cross-shaded.

The complex genetic network of endometriosis inferred by the GeneMANIA bioinformatics tool is given in Figure 3, in which is included information about 34 disorder gene-candidates (functionality-related with the studied SNPs) and 20 high-collaboration genes. We found crucial relationships between these genes with maximum prevalence of collective expression (the contribution share is equal to 76.27%) and high occurrence of physical relationship process (13.17%). A little more than 5% in the endometriosis complex genetic network is allocated to collaborative domain of proteins (5.34%) and common localization (5.23%). Among the high-collaboration genes (Supplementary Table S8), the most substantial rank coefficients had *ARL14EPL* (*ADP ribosylation factor like GTPase 14 effector protein like*), *ATP5MF-PTCD1* (this locus represented both transcript encodes *ATP5J2* (*ATP synthase*, *H+ transporting*, *mitochondrial Fo complex*, *subunit F2*) and *PTCD1* (*pentatricopeptide repeat domain 1*) and *LRPPRC* (*leucine rich pentatricopeptide repeat containing*).

The enrichment analysis of biological pathways underlying endometriosis (investigated by the Gene Ontology tool) allowed us to establish only two significant pathways (biological process and protein class) which included organic anion transport (P_FDR_= 0.0008) and secondary carrier transporter (P_FDR_= 0.005).

## 3. Discussion

The present study was the first to perform a genetic association and in silico functionality analysis aiming to better understand the biology pathways by which the sex hormone polymorphisms proved by GWAS are involved in the molecular pathophysiology of endometriosis. We observed independent protective effects (OR = 0.64–0.68) for endometriosis of the two SNPs located in the *FSHB* chain promoter (rs11031002 T>A and rs11031005 T>C) and an increased disease-risk effect TT haplotype caused by these loci (OR = 2.03). Furthermore, eight GWAS loci of the sex hormone levels within twelve simulation genetic models are causal for the disease. In addition to the GWAS involvement of the studied SNPs in the levels of several sex hormones, according to our in silico data, these 8 loci and 80 proxy SNPs are genome variants affecting expression, splicing, epigenetic and amino acid conformation of the 34 genes.

According to previous GWAS data, and analyzed in the present study, the *FSHB* promoter polymorphisms (rs11031002 and rs11031005) were associated with multiple female reproductive outcomes. For rs11031002, these include LH level (*beta* = 0.221 for allele A) [16]; serum levels of protein CGA; FSHB (*beta* = −0.162 for allele A) [19]; polycystic ovary syndrome (PCOS) (OR = 1.24 for allele A) [20]; and bone mineral density (*beta* = 0.02 for allele T) [21]. For SNP rs11031005, the previous GWAS demonstrated a correlation with both endometriosis and migraine (OR = 1.08 for allele T) [5]; FSH concentration (*beta* = −0.232 for allele C) [16]; testosterone levels—total (*beta* = 0.033 for allele C) and bioavailable (*beta* = 0.023 for allele C) [17]; age at menarche (*beta* = −0.035 for allele T) [22] and age at menopause [23], PCOS (*beta* = −0.159 for allele T) [24]; and ovarian cyst (*beta* = −0.110 for allele C) [25]. The above-mentioned data is evidence of strongly pronounced pleiotropic effects of the *FSHB* chain promoter polymorphisms. Thus, the currently available literature data and the results of our study indicate the presence of FSH-lowering, and LH- and testosterone-heightening, due to the *FSHB* promoter loci (alleles A rs11031002 and C rs11031005) that is strongly associated with low risk of endometriosis. By contrast, a high level of FSH including those caused by genetic polymorphisms (genetically inherited elevated basal levels of FSH) increases the risk of endometriosis. Interestingly, FSH-lowering allelic variants of the *FSHB* promoter (determining the low risk of endometriosis) are correlated with longer menstrual cycles, later age at menopause, and greater female nulliparity; and these female reproductive conditions are also independent protective factors for endometriosis [12].

The *FSHB* gene encodes the β-subunit of FSH which is associated with an α-chain and forms FSH heterodimer (data of GeneCards, https://www.genecards.org/ (accessed on 15 July 2022)). FSH is vital for the hypothalamic-pituitary-gonadal axis and performs a paramount role in human reproductive processes [13]. The FSH production process occurs in the anterior pituitary [12]. FSH binding to the G-protein-coupled receptor (FSHR) promotes follicle development, estrogen production, granulosa cell growth, and androgen-converting enzyme aromatase synthesis induction in women [12,13]. Gonadotropin releasing hormone (GnRH) positively regulates synthesis and secretion of FSH from the pituitary. Estrogen, progesterone, andtestosterone can regulate transcription of the *FSHB* gene by suppressing GnRH (due to the MAPK/ERK phosphorylation pathway) and modulating activin-involved processes (TAK1 pathway with SMAD signal cascade, etc.) [26]. Of note is that high FSH levels (due to direct or indirect effects) may stimulate abnormal growth of endometrium resulting in a higher risk of endometriosis [12]. Interestingly, the *FSHB* gene polymorphisms can be involved in the biology of endometriosis not only due to the above-mentioned FSH pathways, but also due to the effects of *ARL14EP*, the expression of which they control (according to our in silico models, endometriosis high risk alleles T of rs11031002 and rs11031005 are linked with low expression of *ARL14EP*). *ARL14EP* is expressed in a variety of organs/tissues, including ovary, uterus, etc. [11]. The protein encoded by *ARL14EP* (ADP ribosylation factor-like GTPase 14 effector protein) interacts with ARL14 (ADP-ribosylation factor-like 14), ACTB (beta-actin), and MYO1E (actin-based motor protein myosin 1E) and controls the export of MHCII molecules [11]. This assumption requires experimental confirmation in future studies.

According to our data, along with two polymorphisms of the rs11031002 and rs11031005 *FSHB* gene, six more polymorphic loci associated with the level of sex hormones are involved in susceptibility to endometriosis as part of the models of intergenic interactions: these are rs34670419 *ZKSCAN5*, rs112295236 *SLC22A10*, rs117585797 *ANO2*, rs117145500 *CHD9*, rs727428 *SHBG*, and rs1641549 *TP53.* These polymorphic loci are GWAS correlated with circulating levels of sex hormones such as SHBG (rs727428 *SHBG* [14] and rs1641549 *TP53* [16]), oestradiol (rs117585797 *ANO2* [16]), bioavailable testosterone (rs727428 *SHBG* [17] and rs112295236 *SLC22A10* [17]), FAI (rs117145500 *CHD9* [16]), DHEAS (rs34670419 *ZKSCAN5* [15]), progesterone (rs112295236 *SLC22A10* [16] and rs34670419 *ZKSCAN5* [16]), and Cortisol/DHEAS ratio (rs34670419 *ZKSCAN5* [15]). Thus, the levels of sex hormones determined by polymorphisms of candidate genes define the susceptibility to the development of endometriosis. It is known from the literature that specific characteristics of the hormone profile of women with endometriosis are: decreased frequencies/amplitudes of GnRH and LH pulses, resulting reduced LH, increased FSH, decreased LH/FSH ratio, increased SHBG, reduced serum/follicular testosterone, high estradiol concentration in lesions (local) and normal or low level of serum estradiol, increased estradiol/testosterone ratio, change of aromatase expression, etc. [4,7,27]. These hormone alterations predetermine and favor endometrial proliferation, pelvic inflammation, ectopic implants and other endometriosis phenotypic signs [7,28]. It should be noted that the concentrations of sex hormones are largely correlated with each other [16] and therefore their phenotypic effects (including in the development of endometriosis) as a rule are interrelated and interdependent.

The results of the present study are completely consistent with the hypothesis recently referred to in reviews by Dinsdale and Crespi that low prenatal testosterone concentration levels may be a cause of endometriosis development in the later life of a female [4,7,29]. The authors hypothesized that low levels of testosterone in the prenatal period reprograms the hypothalmic–pituitary–gonadal (HPG) axis of the female fetus, resulting in distinct changes of several sex hormone productions such as lower LH relative to FSH, higher SHBG, lower ovarian and serum testosterone, etc., that finally predispose the adult to endometriosis [4,29]. In females, testosterone is produced by ovary and adrenal glands, and possesses significant functionality in muscle tissue and bone growth/density, regulates folliculogenesis and decidualization, promotes storage of energy and inhibits inflammation [7,30]. The hormones of HPG axis (GnRH and LH) are key regulators in the production of testosterone by negative feedback loop [30] and decrease in GnRH and LH pulses resulting in lower levels of testosterone that correlate with high endometriosis risk [7]. Interestingly, nearly 60% of circulating testosterone in females is bound to SHBG (also called non-bioavailable testosterone) [30] and therefore SHBG influences its bioavailability [7]. In the GWAS study, Sinnott-Armstrong et al. found strong opposite genetic correlation between the bioavailable testosterone and SHBG in females (r^2^ = −0.75) [29]. It is important to emphasize that we observed the GWAS-correlated association with SHBG level rs727428 *SHBG* and rs1641549 *TP53* within the epistasis interaction models of endometriosis risk.

To explore whether endometriosis and sex hormone levels have a common genetic origin, Garitazelaia et al. [18] performed a mendelian randomization analysis using public GWAS data of endometriosis (the studied FinnGen cohort included 3380/31753 cases/controls) and data on nine sex hormone levels (identical to our list) [16]. As a result of this analysis, on the one hand the authors did not reveal reliable causal relationships between reproductive hormone levels and endometriosis; on the other hand, what was a surprise for them were the revealed pleiotropic genetic associations of the two loci of the *FSHB* subunit promoter (rs11031002 and rs11031005) with endometriosis and sex hormone levels [18]. In their work, negative *beta*-values for rs11031002 and positive *beta*-values for rs11031005 were found, indicating that these SNP effects were contradictory, whereas in our study, the effects of these loci in endometriosis susceptibility were non opposite and only protective.

Moreover, according to the study of Garitazelaia and co-authors, SNPs (rs11031005 and rs11031006) of the region near to *FSHB* (25–40 Kb upstream) in chromosome 11 showed significant pleiotropic associations mediating endometriosis and correlated traits (age at menopause and age at menarche, and length of menstrual cycle) [18].

Numerous GWAS data confirm the importance of the *FSHB* promoter region SNP rs11031006 for several female reproductive outcomes including age at menarche [31] and menopause [32]; menstrual cycle parameters (menstrual cycle length and excessive, frequent and irregular menstruation) and bilateral oophorectomy [33]; dizygotic twinning and FSH concentration [34]; uterine fibroids [35]; and PCOS, FSH and LH concentrations [36,37]. In this context, Ruth et al. showed that a SNP rs10835638 in the same region is associated with low FSH levels, longer menstrual cycles, later age at menopause, greater female nulliparity and lower risk of endometriosis [12]; and Bianco et al. demonstrated the effect of this locus on LH concentration in infertile women with endometriosis [13]. Genetic variant −211 G>T *FSHB* (rs10835638) was determinant of serum FSH and LH levels in eumenorrheic healthy and infertile women [38], and associated with the response to controlled ovarian hyperstimulation, LH concentration, antral follicle counting, oocytes retrieval and embryos [39], and PCOS [40]. Other studies also reported the association of the *FSHB* promoter polymorphisms with endometriosis (rs74485684) [6], menarcheal age (rs11031010, rs1782507, rs555621) [41], PCOS and LH level in patients (rs11031010) [42], and menopause age (rs12294104) [43]. Previously, we reported the SNP (rs11031010, rs1782507, rs555621) association in the same region as the *FSHB* gene (8–16 Kb upstream) and in the same cohorts of woman with endometriosis [10], uterine leiomyoma [44], endometrial hyperplasia [45] and BMI (rs555621) [46]. Thus, the above-mentioned literature data indicate that common highly correlated polymorphisms situated in a functional activity region (promoter) upstream of the *FSHB* gene controls FSH, LH and testosterone levels in a woman’s organism, with subsequent strongly pronounced multiple effects on several reproductive traits/diseases. The key role of the *FSHB* promoter region on genetic variants for female reproductive outcomes is noted in the reviews by Gajbhiye et al. [47], McGrath et al. [11] and Dinsdale et al. [4].

According to the results of the present study, a significant contribution to endometriosis susceptibility, along with the main effects of two polymorphisms (rs11031002 and rs11031005) of the *FSHB* gene, is also made by polymorphisms rs117585797 of the *ANO2* gene within the three intergenic interaction models. SNP rs117585797 localized in the intron of the *ANO2* gene is GWAS-significant for the estradiol level [16] in accordance with the data obtained by us in silico, and has an impact on TF-regulatory effects (influencing the affinity of the regulatory DNA motif to transcription factors Crx and Gsc). *ANO2* (*anoctamin 2*) also known as *TMEM16B* (*transmembrane protein 16B*) refers to a family of calcium-activated chloride channels (CaCCs). This gene is a protein coding and is involved in the processes of the protein homodimerization activity and pathway of the intracellular CaCCs activity (GeneCards data). There are experimental data on the expression of the *ANO2* (together with the *ANO1* gene with which the *ANO2* closely interacts) in human and murine myometrial tissue [48]. It is assumed that *ANO2/1* are functionally related to intracellular calcium flow both through the calcium channels of the cell membrane and through intracellular release from the sarcoplasmic reticulum [48]. It should be noted that despite the fact that the rs117585797 *ANO2* polymorphism, together with the rs11031002 and rs11031005 polymorphisms, is part of the most significant models of intergenic interactions associated with endometriosis, as a result of bioinformatic analysis (using Gene Ontology and GeneMANIA software), we have not revealed convincing evidence of the exact mechanisms of these interactions. Based on this, it can be assumed that there are some complicated mediated mechanisms of interaction of these genes that underlie their common risk effect on endometriosis, and these pathways will have to be revealed in future studies. At the same time, significant phenotypic effects of the interaction of these polymorphisms with respect to endometriosis can manifest themselves in the interaction of sex hormones whose levels they determine, such as LH [16], serum levels of protein CGA, FSHB [19], (rs11031002 *FSHB*), FSH [16], total and bioavailable testosterone levels [17], (rs11031005 *FSHB*), and oestradiol (rs117585797 *ANO2* [16]). As we have indicated above, pronounced interrelated changes in the concentrations of sex hormones and the ratio of their levels (reduced LH, increased FSH, decreased LH/FSH ratio, reduced testosterone, increased estradiol/testosterone ratio, etc. [4,7,27]) play a decisive role in the disease pathophysiology.

We found that the locus of the *ZKSCAN5* gene (rs34670419) in the composition of three multi-level (2,3,4 loci) most noteworthy intergenic interactions models is associated with endometriosis. Polymorphism rs34670419 shows a pronounced effect (GWAS data) in determining the concentration/ratio of several hormones such as DHEAS [15], progesterone [16]), and Cortisol/DHEAS ratio [15]. Also this polymorphism is GWAS correlated with urinary metabolite levels, including steroid conjugates (16a-hydroxy DHEA 3-sulfate, andro-steroid-monosulfate) [49]. Several polymorphisms are in linkage disequilibrium (r^2^ = 0.62–1.00) with rs34670419 *ZKSCAN5* and GWAS involved in endometriosis-related phenotypes such as DHEAS (rs10278040 [50], rs11761528 [51]), blood metabolite (andro-sterone sulfate, etc,) (rs10278040 [52], rs12533251 [53]), testosterone (rs11761528 [54]), BMI (rs11761528 [55], rs3901286 [23]). Our in silico data show a functional association of rs34670419 *ZKSCAN5* (SNP is located in the 3’-UTR region of this gene) with seven genes such as *ZKSCAN5*, *CYP3A7*, *GS1-259H13.2*, *OR2AE1*, *PTCD1*, *TRIM4*(eQTL) and *GPC2* (sQTL). *ZKSCAN5* (*zinc finger with KRAB and SCAN domains 5*) encodes a zinc finger protein ZFP-95 (Kruppel family) which may be involved in transcriptional regulation (GeneCards data). The above-mentioned GWAS data indicate pronounced links of *ZKSCAN5* gene polymorphic variants and strongly coupled SNPs with differences in sex hormones and their conjugates (metabolites); the potential mechanisms of these connections remain unknown [51], however, and require further detailed research. *TRIM4* is a member of the tripartite motif family of genes. The protein encoded by this gene (tripartite motif containing 4) contains three zinc-binding domains, is localized in cytoplasm and has unidentified function (GeneCards data). The *CYP3A7* is a member of the cytochrome P450 superfamily and is implicated in the synthesis processes of cholesterol, steroids such as hydroxylates testosterone and dehydroepiandrosterone 3-sulphate (GeneCards information). The *CYP3A7* gene affects the metabolism of endogenous sex hormones (oestrone and progesterone) in premenopausal women, and due to this, it can determine breast cancer risk [56].

We established the involvement in the endometriosis biology of SNP rs112295236 *SLC22A10* within three multi-tiered (2,3,4 loci) most worthwhile intergenic epistatic models. There are compelling GWAS data on the correlation of this polymorphism with the level of bioavailable testosterone [17] and progesterone [16]. Interestingly, polymorphisms rs112295236 is strongly linked (r^2^ = 0.87–1.00) to the two locii (rs1939769 and rs113172275) which are GWAS-correlated with the concentration of total [57] and bioavailable [17] testosterone in women. The in silico results obtained in this work indicate the functional relationship (epigenetic and eQTL) of this polymorphism (locus is situated in site 3.7kb 5’ of *SLC22A24*) and 65 SNPs linked with it with the five genes *SLC22A24*, *SLC22A9*, *SLC22A10*, *SLC22A25*, *ATL3.* At the moment it is known that *SLC22A24* is transporter of organic ions across the cell membranes (GeneCards data) with no known ligands and not fully known biological functions/pathways [58]. Based on cellular and computational methods, Yee et al. [58] have shown a significant role of *SLC22A24* in transport conjugates of steroid hormones (in particular, conjugated steroid reabsorption in kidney), bile acids, and other dicarboxylic acids, which indicates the key role of *SLC22A24* in dysregulated metabolism of steroid. Given the great importance of the *SLC22A24* in the in processes of steroid homeostasis, reasoned assumptions are made about the use of this gene as a pharmacological target for regulating levels of steroid [58]. The *SLC22A10*, *SLC22A25*, *SLC22A9* also belong to the superfamily of human solute carrier transporters and have no known appointed ligands (the so-called orphan transporters) [58]. It is important to note that there are literature data (GWAS) on the relationship of several polymorphic loci located in these gene regions with levels of total testosterone (rs143088266 [17]) and estriol (rs184061227, rs117070489 and rs511686 [59]).

It should be noted that, if confirmed in other independent studies, the data obtained in our study on the strongly associated FSH-heightening, and LH- and testosterone-lowering of the *FSHB* promoter loci (TT haplotype of the rs11031002 and rs11031005) with a higher risk of endometriosis may in the future (find application in clinical practice. In particular, the above-mentioned polymorphisms of the *FSHB* gene promoter region could become quite effective biomarkers that would make it possible to identify a group of women at higher risk of developing endometriosis, and to carry out measures for reducingthe chances of the disease developing and for its early diagnosis. The prospects of using *FSHB* promoter polymorphism (−211 G>T *FSHB*, rs10835638) in practical medicine (in fertility clinics) to identify patients with genetically inherited elevated basal levels of FSH and LH are also indicated in previous work [38]. Clearly, at this point in time, there remains “an urgent unmet need to identify novel clinical markers of endometriosis” [3] and “future research must focus on understanding the pathogenesis, …, developing non-invasive diagnostic methods” [1].

The limitation of this study is the lack of experimental confirmation of the relationships between the *FSHB* subunit promoter SNPs (rs11031002 and rs11031005) associated with endometriosis according to the results obtained by us with the level of appropriate sex hormones (LH, FSH, testosterone level, etc.) in women in the studied samples of patients and controls. Moreover, another limitation of this study is the need for experimental proof of the functional effects of endometriosis-significant SNPs that we have identified in silico.

## 4. Materials and Methods

### 4.1. Study Subjects

The study design was approved by the independent Human Research Ethics Committee of the “Belgorod State University”. All experiments were performed in accordance with relevant guidelines and regulations. Each patient enrolled in this study signed an informed consent form for all procedures and to allow clinical examination data and biological sample collection and analysis for research purposes. A total number of 1376 women (395 endometriosis patients and 981 controls) were recruited as participants at the Perinatal Center, Belgorod Regional Hospital of St. Joasaph 2008–2013 in the framework. All participants of this study were of Russian European ancestry and birthplace/living in Central Russia [60,61]. Women with a history of female reproductive organ cancer, severe autoimmune or vital organ disorder were not eligible for recruitment [10].

Diagnosis of endometriosis is verified by experienced gynecologists and confirmed by clinical and instrumental examination methods (laparotomy or laparoscopy and subsequent morphological evidence). According to the rASRM classification [62], stage I (35.90%), II (53.98%) and III/IV (10.12%) have been identified among 395 endometriosis patients. Women without clinical symptoms (chronic pelvic pain, etc.) and ultrasound data for disorders of the female reproductive organs (small pelvis) were control group. Table 4 summarizes the demographic, reproductive and gynecological characteristics/pathologies of the case study and control participants, which were present in previous endometriosis genetic studies conducted in the same sample of patients and controls [10]. The endometriosis females as compared to control had shorter menstrual cycle length and lower parity, increased number of medical abortions in the anamnesis, higher percentage of history of infertility, pelvic organ surgery (laparoscopy/laparotomy), family history of the disorder (Table 4), and these endometriosis risk factors were included in all genetic association tests (logistic regression models and MB-MDR analyses) as covariates [10].

### 4.2. Laboratory Examination of SNPs

DNA samples were obtained from the biobank of the Department of Medical Biological Disciplines of Belgorod State University. The samples were collected previously in the context of endometriosis genetic studies for the period from 2008 to 2013 [10].

Polymorphic variants of genes correlated with sex hormone levels (prevalence) in women (≈90%) at a genome-wide significance (*p* ≤ 5 × 10^−8^) (Supplementary Table S9), and demonstrated functional significance (according to HaploReg internet resources [63]) were selected for the present study [64,65] (Supplementary Table S10). In total, nine SNPs were included in our study, namely, rs148982377 *ZNF789*, rs34670419 *ZKSCAN5*, rs11031002 and rs11031005 *FSHB*, rs112295236 *SLC22A10*, rs117585797 *ANO2*, rs117145500 *CHD9*, rs727428 *SHBG*, and rs1641549 *TP53.* These GWAS correlated with circulating levels of sex hormones: sex hormone-binding globulin (SHBG), oestradiol, total and bioavailable testosterone, free androgen index (FAI) ((testosterone/SHBG) × 100), dehydroepiandrosterone sulphate (DHEAS), follicle-stimulating hormone (FSH), luteinizing hormone (LH), progesterone, and Cortisol/DHEAS ratio (Supplementary Table S9) [14,15,16,17].

SNPs were genotyped by PCR and TaqMan allelic discrimination assay on the CFX96 detection system (Bio-Rad Laboratories, USA) [66]. Genotyping quality control was guaranteed by inclusion ≈5% blind case/control DNA replicates and theirs regenotyping [67].

### 4.3. Genetic Data Statistical Analysis

Fisher’s exact test was used for analysis of the corresponding genotype distribution of the genetic variants in question relative to the Hardy–Weinberg equilibrium [68].

Additive, dominant, recessive, allelic genetic models and logistic regression procedure were tried for recognition of endometriosisgenotype associations. Parameters of odds ratio (OR) and 95% confidence intervals (95% CI) were employed for measurements of the association of alleles/genotypes/haplotypes with the disorder [69,70,71]. In accordance with data from previous endometriosis genetic studies [10], in these cohorts (cases/controls) of female participants, association parameters were adjusted for covariates such asparity, length of menstrual cycle, history and number of medical abortions, infertility, and pelvic surgery (Table 4). In order to adjust for multiple testing, the permutation procedure was applied [72,73]. Accepted as statistically significant for each SNP and their haplotypes were the P_permutation_ values < 0.0125 and <0.007: Bonferroni threshold to correct for multiple testing estimated at 0.05/4 (considering the number of genetic models—four) and 0.05/7 (adjustment for multiple comparisons for the number of haplotypes studied—seven—for two pair of high linked SNPs (rs148982377–rs34670419, r^2^ = 1.00, and rs11031002–rs11031005, r^2^ = 0.79) using the Bonferroni correction), respectivly [74]). All genetic association and permutation tests were implemented in PLINK software (version 1.07) [75].

MB-MDR statistical software [76] for R package was used for evaluating the phenotypic effects of the considered SNPs/genes interactions on endometriosis [77]. MB-MDR analyses were adjusted for appropriate cofactors and multiple testing procedures, analogously to the individual SNP association tests. For the permutation test, we selected genetic interaction models which corresponded to the following P (Wald’s test) values for several N-way models (having calculated the Bonferroni threshold for the numbers of N-way combinations of the 9 considered SNPs): 2-way—*p* < 1.38 × 10^−3^ (0.05/36), 3-way—*p* < 5.95 × 10^−4^ (0.05/84) and 4-way—*p* < 3.97 × 10^−4^ (0.05/126). The P_permutation_ value < 0.01 was adopted as statistically significant.

### 4.4. In Silico SNP Analysis

For the purposes of a detailed assessment of the SNP-gene, prediction functions were applied in silico methodology [78,79,80] and several tissue/organ-specific epigenetic and gene expression/splicing databases were employed (HaploReg [63] and GTExproject [81], respectively) and some other publicly available bioinformatics online resources (PolyPhen-2 [82], Gene Ontology [83], STRING [84], GeneMANIA [85]). In order to study in depth the functional effects of endometriosis susceptibility loci, we also evaluated the functionality of high linked SNPs (r^2^ ≥ 0.8) [86,87].

## 5. Conclusions

The sex hormone polymorphisms proven by GWAS are associated with endometriosis risk (independently, within haplotypes and interlocus relationships) and are involved in the molecular pathophysiology of the disease by the effect of the resulting expression, splicing, epigenetic and amino acid conformation of the more than thirty genes.

## Figures and Tables

**Table 1:** Associations of the studied gene polymorphisms with endometriosis.

Chr	SNP	Minor allele	Gene	n	Allelic model				Additive model				Dominant model				Recessive model			
					OR	95% CI		*p*	OR	95% CI		*p*	OR	95% CI		*p*	OR	95% CI		*p*
						L95	U95			L95	U95			L95	U95			L95	U95	
7	rs148982377	C	*ZNF789*	1329	1.21	0.84	1.75	0.311	1.25	0.84	1.85	0.262	1.25	0.83	1.87	0.282	2.16	0.13	34.74	0.587
7	rs34670419	T	*ZKSCAN5*	1329	1.14	0.75	1.72	0.545	1.18	0.77	1.82	0.437	1.20	0.77	1.89	0.421	1.08	0.10	2.04	0.952
11	rs11031002	A	*FSHB*	1309	**0.68**	**0.52**	**0.90**	**0.006**	**0.64**	**0.47**	**0.85**	**0.003**	**0.60**	**0.43**	**0.82**	**0.002**	0.73	0.24	2.21	0.580
11	rs11031005	C	*FSHB*	1330	**0.66**	**0.50**	**0.87**	**0.003**	**0.65**	**0.49**	**0.87**	**0.004**	**0.65**	**0.49**	**0.92**	**0.009**	0.17	0.03	0.78	0.023
11	rs112295236	G	*SLC22A10*	1321	1.29	0.90	1.84	0.159	1.40	0.96	2.04	0.078	1.04	0.95	2.07	0.093	2.52	0.31	20.17	0.384
12	rs117585797	A	*ANO2*	1312	0.87	0.50	1.52	0.622	0.98	0.55	1.75	0.947	0.997	0.55	1.80	0.991	0.01	0	inf	0.999
16	rs117145500	C	*CHD9*	1311	1.18	0.90	1.55	0.220	1.13	0.85	1.51	0.398	1.11	0.81	1.52	0.503	1.81	0.54	6.06	0.332
17	rs727428	T	*SHBG*	1321	0.95	0.80	1.13	0.555	0.96	0.80	1.15	0.652	0.89	0.69	1.15	0.367	1.07	0.75	1.54	0.701
17	rs1641549	T	*TP53*	1312	0.89	0.73	1.09	0.250	0.89	0.72	1.09	0.266	0.83	0.64	1.08	0.164	0.99	0.60	1.65	0.979

Note: OR—odds ratio; 95% CI—95% confidence interval; all results were obtained after adjustment for covariates; P_perm_ values < 0.0125 are shown in bold.

**Table 2:** Associations of the gene-candidate haplotypes of sex hormone level with endometriosis.

Haplotypes	Frequency		OR	P	P_perm_
	Endometriosis Patients (*n* = 395)	Controls (*n* = 981)			
rs148982377 *ZNF789*–rs34670419 *ZKSCAN5*					
CT	0.040	0.035	1.22	0.402	-
CG	0.017	0.013	1.26	0.536	-
TG	0.943	0.952	0.80	0.238	-
rs11031002–rs11031005 *FSHB*					
AC	0.087	0.113	0.87	0.360	-
TC	0.007	0.023	0.16	0.0002	0.001
AT	0.008	0.022	0.09	0.000001	0.001
TT	0.898	0.842	2.03	0.000002	0.001

Note: OR—odds ratio; p—significance level; the results were obtained by the logistic regression analysis with adjustment for covariates.

**Table 3:** SNP × SNP interactions significantly associated with endometriosis.

N	SNP × SNP Interaction Models	NH	*Beta* H	WH	NL	*Beta* L	WL	p_perm_
Two-order interaction models (*p* < 6.52 × 10^−4^)								
1	rs11031002 *FSHB* × rs11031005 *FSHB*	1	0.741	22.24	2	−2.002	32.68	<0.001
2	rs11031002 *FSHB* × rs112295236 *SLC22A10*	1	0.288	4.23	1	−0.629	12.95	0.001
3	rs117145500 *CHD9* × rs11031002 *FSHB*	1	0.281	4.43	1	−0.660	11.68	0.003
4	rs11031002 *FSHB* × rs34670419 *ZKSCAN5*	1	0.386	7.04	1	−0.594	11.62	0.004
5	rs11031002 *FSHB* × rs727428 *SHBG*	0	-	-	1	−0.850	12.15	0.009
Three-order interaction models (*p* < 5.01 × 10^−8^)								
1	rs11031002 *FSHB* − rs117585797 *ANO2* × rs11031005 *FSHB*	1	0.672	20.33	2	−2.377	36.04	<0.001
2	rs11031002 *FSHB* − rs112295236 *SLC22A10 −* rs11031005 *FSHB*	1	0.489	12.49	2	−2.502	35.58	<0.001
3	rs11031002 *FSHB* − rs1641549 *TP53* × rs11031005 *FSHB*	1	0.410	10.51	5	−1.975	31.58	<0.001
4	rs11031002 *FSHB* − rs11031005 *FSHB* − rs34670419 *ZKSCAN5*	1	0.582	16.53	2	−2.003	29.91	<0.001
5	rs117145500 *CHD9 −* rs11031002 *FSHB* − rs11031005 *FSHB*	1	0.408	9.50	3	−2.001	29.71	<0.001
Four-order interaction models (*p* < 8.20 × 10^−9^)								
1	rs11031002 *FSHB* − rs117585797 *ANO2 −* rs112295236 *SLC22A10* − rs11031005 *FSHB*	1	0.464	11.86	2	−2.884	35.70	<0.001
2	rs11031002 *FSHB* − rs117585797 *ANO2 −* rs11031005 *FSHB* − rs34670419 *ZKSCAN5*	1	0.560	16.26	3	−2.425	33.22	< 0.001

Note: NH—number of significant high risk genotypes in the interaction; beta H—regression coefficient for high risk exposition in the step 2 analysis; WH—Wald statistic for high risk category; NL—number of significant low risk genotypes in the interaction; beta L—regression coefficient for low risk exposition in the step 2 analysis; WL—Wald statistic for low risk category; p_perm_—permutation *p*-value for the interaction model (1.000 permutations); the results were obtained using the MB-MDR method with adjustment for covariates.

**Table 4:** Characteristics of participants from the case and control groups.

Parameters	Cases (*n* = 395) $\bar{X}$ ± SD/% (*n*)	Controls (*n* = 981) $\bar{X}$ ± SD/% (*n*)	*p*
Age, years	39.75 ± 9.01	40.73 ± 8.60	>0.05
Height, m	1.65 ± 0.06	1.65 ± 0.06	>0.05
Weight, kg	72.65 ± 14.38	72.49 ± 13.37	>0.05
BMI, kg/m^2^	26.63 ± 5.31	26.66 ± 4.61	>0.05
Proportion of the participants by relative BMI, % (n):			
underweight (<18.50)	4.30 (17)	1.12 (11)	
normal weight (18.50–24.99)	37.72 (149)	42.41 (416)	
overweight (25.00–29.99)	31.65 (125)	30.49 (299)	>0.05
obese (>30.00)	26.33 (104)	25.99 (255)	
Family history of endometriosis (yes)	6.07 (24)	1.94 (19)	**<0.001**
Married	82.53 (326)	85.93 (843)	>0.05
Smoking (yes)	18.22 (72)	17.33 (170)	>0.05
Drinking alcohol (≥7 drinks per week)	4.05 (16)	3.06 (30)	>0.05
History of pelvic surgery (laparoscopy and/or laparotomy)	15.19 (60)	9.99 (98)	**<0.01**
Oral contraceptive use	8.10 (32)	10.09 (99)	>0.05
Age at menarche and menstrual cycle			
Age at menarche, years	13.29 ± 1.27	13.27 ± 1.25	>0.05
Proportion of the participants by relative age at menarche,% (n)			>0.05
early (<12 years)	6.36 (25)	6.42 (63)	
average (12–14 years)	81.17 (319)	79.51 (780)	
late (>14 years)	12.47 (49)	14.07 (138)	
Duration of menstrual bleeding (mean, days)	5.13 ± 1.56	4.94 ± 0.94	>0.05
Menstrual cycle length (mean, days)	27.66 ± 2.28	28.15 ± 2.24	**<0.001**
Reproductive characteristic			
Age at first birth (mean, years)	21.25 ± 3.04	21.71 ± 3.49	>0.05
No. of gravidity (mean)	2.60 ± 2.31	2.45 ± 1.55	>0.05
No. of births (mean)	1.07 ± 0.97	1.51 ± 0.67	**<0.001**
No. of spontaneous abortions (mean)	0.21 ± 0.61	0.24 ± 0.51	>0.05
No. of induced abortions (mean)	1.25 ± 1.61	0.67 ± 0.99	**<0.001**
No. of induced abortions:			**<0.001**
0	46.58 (184)	58.92 (578)	
1	17.22 (68)	23.75 (233)	
2	19.24 (76)	10.40 (102)	
3	8.61 (34)	5.40 (53)	
≥4	8.35 (33)	1.53 (15)	
History of infertility	32.42 (132)	5.20 (51)	**<0.001**
Gynecological pathologies			
Uterine leiomyoma	52.40 (207)	-	-
Endometrial hyperplasia	46.33 (183)	-	-
Adenomyosis	43.04 (170)	-	-

## Data Availability

The data generated in the present study are available from the corresponding author upon reasonable request.

## Supplementary Materials

The following supporting information can be downloaded at: https://www.mdpi.com/article/10.3390/ijms232213691/s1.
