# Supplementary material for: Sex Hormone Candidate Gene Polymorphisms Are Associated with Endometriosis
Source: Int J Mol Sci. 2022 Nov 8;23(22):13691. doi: 10.3390/ijms232213691 (PMC9697627; doi:10.3390/ijms232213691)
Supplement: Supplementary file 1 [file ijms-23-13691-s001.zip › Suppl table S1.pdf]

Supplementary Table S1

The allele and genotype frequencies of the studied SNPs in the endometriosis and control groups

Chr	SNP	Gene	Minor allele	Major allele	Minor allele frequency	Number of the studied chromosomes	Genotype distribution*	H_o	H_e	P_{HWE}
Endometriosis patients (n=395)										
7	rs148982377	*ZNF789*	C	T	0.057	770	1/42/342	0.109	0.108	1.000
7	rs34670419	*ZKSCAN5*	T	G	0.044	768	1/32/351	0.083	0.085	0.534
11	rs11031002	*FSHB*	A	T	0.096	756	4/65/309	0.172	0.174	0.766
11	rs11031005	*FSHB*	C	T	0.094	766	2/68/313	0.177	0.170	0.557
11	rs112295236	*SLC22A10*	G	C	0.064	768	2/45/337	0.117	0.119	0.659
12	rs117585797	*ANO2*	A	C	0.022	758	0/17/362	0.045	0.044	1.000
16	rs117145500	*CHD9*	C	A	0.113	760	5/76/299	0.200	0.201	1.000
17	rs727428	*SHBG*	T	C	0.372	760	54/175/151	0.460	0.467	0.826
17	rs1641549	*TP53*	T	C	0.220	764	26/116/240	0.304	0.343	0.026
Control group (n=981)										
7	rs148982377	*ZNF789*	C	T	0.048	1888	1/88/855	0.093	0.091	0.718
7	rs34670419	*ZKSCAN5*	T	G	0.039	1890	2/70/873	0.074	0.075	0.650
11	rs11031002	*FSHB*	A	T	0.135	1862	17/218/696	0.234	0.234	1.000
11	rs11031005	*FSHB*	C	T	0.136	1894	20/217/710	0.229	0.235	0.488
11	rs112295236	*SLC22A10*	G	C	0.050	1874	2/90/845	0.096	0.095	1.000
12	rs117585797	*ANO2*	A	C	0.026	1866	1/46/886	0.049	0.050	0.462
16	rs117145500	*CHD9*	C	A	0.097	1862	7/167/757	0.179	0.175	0.707
17	rs727428	*SHBG*	T	C	0.385	1882	132/460/349	0.489	0.473	0.335
17	rs1641549	*TP53*	T	C	0.241	1860	61/326/543	0.350	0.366	0.209

Note: * minor allele homozygotes / heterozygotes / major allele homozygotes
