# Supplementary material for: Sex Hormone Candidate Gene Polymorphisms Are Associated with Endometriosis
Source: Int J Mol Sci. 2022 Nov 8;23(22):13691. doi: 10.3390/ijms232213691 (PMC9697627; doi:10.3390/ijms232213691)
Supplement: Supplementary file 1 [file ijms-23-13691-s001.zip › Suppl table S2.pdf]

Supplementary Table S2

Genotype combinations associated with endometriosis *

N	Genotype combinations	*beta*	P	Risk, High/Low
Two-order interaction models				
1	rs11031002 TT x rs11031005 TT	0.74	0.000002	H
	rs11031002 TA x rs11031005 TT	-2.36	0.000002	L
	rs11031002 TT x rs11031005 TC	-1.42	0.001	L
2	rs11031002 TA x rs112295236 CC	-0.63	0.0003	L
	rs11031002 TT x rs112295236 CC	0.29	0.039	H
3	rs117145500 AA x rs11031002 TT	0.28	0.037	H
	rs117145500 AA x rs11031002 TA	-0.66	0.0006	L
4	rs11031002 TA x rs34670419 GG	-0.59	0.0006	L
	rs11031002 TT x rs34670419 GG	0.39	0.008	H
5	rs11031002 TA x rs727428 CT	-0.85	0.0005	L
Three-order interaction models				
1	rs11031002 TT x rs117585797 CC x rs11031005 TT	0.67	0.000006	H
	rs11031002 TA x rs117585797 CC x rs11031005 TT	-2.61	0.000002	L
	rs11031002 TT x rs117585797 CC x rs11031005 TC	-1.91	0.0008	L
2	rs11031002 TA x rs112295236 CC x rs11031005 TT	-3.35	0.000006	L
	rs11031002TT x rs112295236 CC x rs11031005 TT	0.49	0.0004	H
	rs11031002 TT x rs112295236 CC x rs11031005 TC	-1.61	0.002	L
3	rs11031002 TT x rs1641549 CC x rs11031005 TT	0.41	0.001	H
	rs11031002 TA x rs1641549 CC x rs11031005 TT	-3.06	0.003	L
	rs11031002 TA x rs1641549 CT x rs11031005 TT	-1.97	0.003	L
	rs11031002 TT x rs1641549 CC x rs11031005 TA	-1.31	0.032	L
4	rs11031002 TA x rs11031005 TT x rs34670419 GG	-2.24	0.000008	L
	rs11031002 TT x rs11031005 TT x rs34670419 GG	0.58	0.00005	H
	rs11031002 TT x rs11031005 TC x rs34670419 GG	-1.56	0.003	L
5	rs117145500 AA x rs11031002 TT x rs11031005 TT	0.41	0.002	H
	rs117145500 AA x rs11031002 TA x rs11031005 TT	-2.33	0.00003	L
	rs117145500 AA x rs11031002 TT x rs11031005 TC	-1.31	0.016	L
	rs117145500 AC x rs11031002 TA x rs11031005 TT	-2.39	0.034	L
Four-order interaction models				
1	rs11031002 TA x rs117585797 CC x rs112295236 CC x rs11031005TT	-3.35	0.000006	L
	rs11031002 TT x rs117585797 CC x rs112295236 CC x rs11031005TT	0.46	0.0006	H
2	rs11031002 TA x rs117585797 CC x rs11031005 TT x rs34670419 GG	-2.49	0.000006	L
	rs11031002 TT x rs117585797 CC x rs11031005 TT x rs34670419 GG	0.56	0.00005	H
	rs11031002 TT x rs117585797 CC x rs11031005 TC x rs34670419 GG	-2.18	0.0007	L

* Genotype combinations are derived from the interaction models obtained by the MB-MDR method and described in tables 3
