# Supplementary material for: Sex Hormone Candidate Gene Polymorphisms Are Associated with Endometriosis
Source: Int J Mol Sci. 2022 Nov 8;23(22):13691. doi: 10.3390/ijms232213691 (PMC9697627; doi:10.3390/ijms232213691)
Supplement: Supplementary file 1 [file ijms-23-13691-s001.zip › Suppl table S9.pdf]

Supplementary Table S9

The GWAS data about associations of the studied candidate genes polymorphisms with the level of sex hormones in women or prevalence of women

SNP	Position (chr: hg38)	Phenotype	Association (significance) (affected allele)	Reference
rs148982377	7: 99477415	DHEAS	β = -0.255 (p = 1.82 × 10^{-14}) (C)	Ruth K.S. et al., 2016a
rs34670419	7: 99533211	Progesterone	β = -0.346 (p = 6.09 × 10^{-14}) (T)	Ruth K.S. et al., 2016a
		DHEAS	β = -0.780 (p = 2.07 × 10^{-9}) (T)	Wood A.R. et al., 2013
		Cortisol/ DHEAS ratio	β = 0.721 (p = 2.35 × 10^{-8}) (T)	Wood A.R. et al., 2013
rs11031002	11: 30193714	LH	β = 0.221 (p = 3.94 × 10^{-9}) (A)	Ruth K.S. et al., 2016a
rs11031005	11: 30204809	FSH	β = -0.232 (p = 1.74 × 10^{-8}) (C)	Ruth K.S. et al., 2016a
		Total testosterone	β = 0.033 (p = 7.2 × 10^{-17}) (C)	Ruth K.S. et al., 2020
		Bioavailable testosterone	β = 0.023 (p = 1.5 × 10^{-10}) (C)	Ruth K.S. et al., 2020
rs112295236	11: 63147874	Progesterone	β = 0.255 (p = 7.68 × 10^{-12}) (G)	Ruth K.S. et al., 2016a
		Bioavailable testosterone (in men and women combined)	β = 0.031 (p = 2.3 × 10^{-14}) (G)	Ruth K.S. et al., 2020
rs117585797	12: 5902324	Oestradiol	β = 0.624 (p = 1.63 × 10^{-8}) (A)	Ruth K.S. et al., 2016a
rs117145500	16: 52913718	FAI	β = -0.276 (p = 1.50 × 10^{-8}) (C)	Ruth K.S. et al., 2016a
rs727428	17: 7634474	SHBG	β = -0.126 (p = 2.09 × 10^{-16}) (T)	Prescott J. et al., 2012
		Bioavailable testosterone	β = 0.095 (p = 8.3 × 10^{-309}) (T)	Ruth K.S. et al., 2020
rs1641549	17: 7671457	SHBG	β = -0.127 (p = 1.21 × 10^{-15}) (T)	Ruth K.S. et al., 2016a

Abbreviations: Chr, chromosome; DHEAS, dehydroepiandrosterone sulphate; FAI, free androgen index ((testosterone/SHBG) × 100); FSH, follicle-stimulating hormone; LH, luteinizing hormone; SHBG, sex hormone binding globulin.

References

1. Ruth KS, Campbell PJ, Chew S, Lim EM, Hadlow N, Stuckey BG, Brown SJ, Feenstra B, Joseph J, Surdulescu GL, Zheng HF, Richards JB, Murray A, Spector TD, Wilson SG, Perry JR. Genome-wide association study with 1000 genomes imputation identifies signals for nine sex hormone-related phenotypes. Eur J Hum Genet. 2016 Feb;24(2):284-90. doi: 10.1038/ejhg.2015.102.
2. Prescott J, Thompson DJ, Kraft P, Chanock SJ, Audley T, Brown J, Leyland J, Folkerd E, Doody D, Hankinson SE, Hunter DJ, Jacobs KB, Dowsett M, Cox DG, Easton DF, De Vivo I. Genome-wide association study of circulating estradiol, testosterone, and sex hormone-binding globulin in postmenopausal women. PLoS One. 2012;7(6):e37815. doi: 10.1371/journal.pone.0037815.
3. Wood AR, Perry JR, Tanaka T, et al. Imputation of variants from the 1000 Genomes Project modestly improves known associations and can identify low-frequency variant-phenotype associations undetected by HapMap based imputation. *PLoS One*. 2013;8(5):e64343. doi:10.1371/journal.pone.0064343
4. Ruth KS, Day FR, Tyrrell J, et al. Using human genetics to understand the disease impacts of testosterone in men and women. *Nat Med*. 2020;26(2):252-258. doi:10.1038/s41591-020-0751-5
